# Supplementary material for: Self-activating anti-infection implant
Source: Nat Commun. 2021 Nov 25;12:6907. doi: 10.1038/s41467-021-27217-4 (PMC8617033; doi:10.1038/s41467-021-27217-4)
Supplement: Supplementary file 1 — Supplementary Information [file 41467_2021_27217_MOESM1_ESM.pdf]

Supplementary Information

Self-activating anti-infection implant

Fu *et al.*

1-Supplementary figures:

Supplementary Figs. 1-19

Method for MIC determination of HA/MoS_2 against *S. aureus* is related to Supplementary Fig. 10.

Method for MBC determination of *S. aureus* is related to Supplementary Fig. 11.

2-Supplementary table:

Supplementary Table 1

1-SUPPLEMENTARY FIGURES

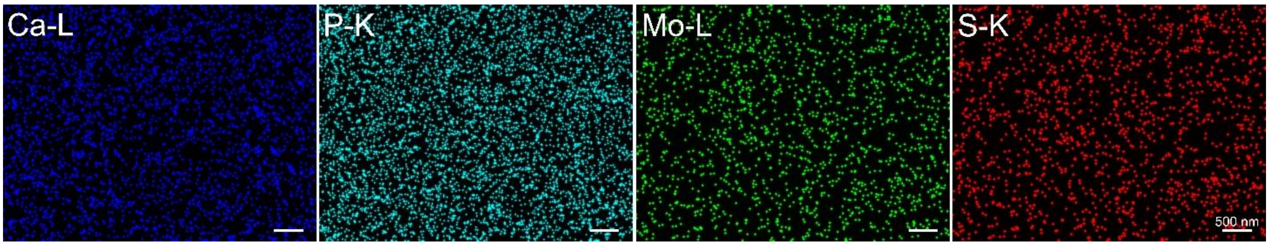

Supplementary Fig. 1 Elemental mapping images of HA/MoS_2-Ti6 in **Fig. 2b**. (n=3 independent samples).

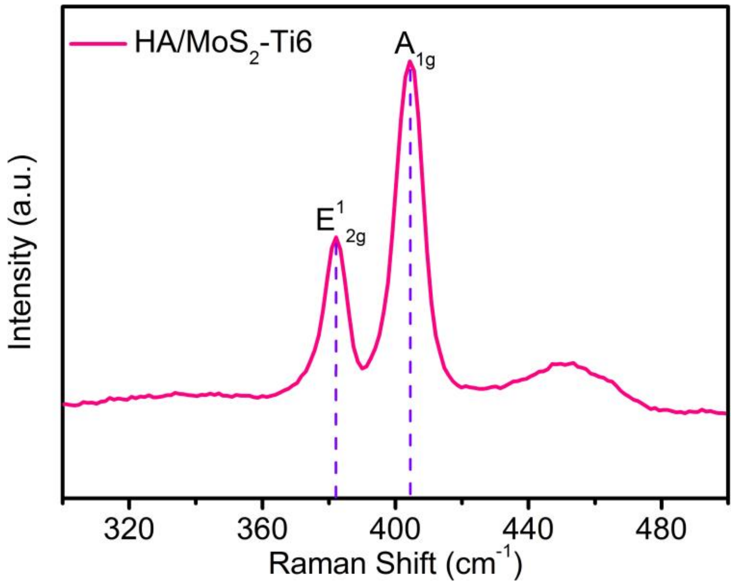

Supplementary Fig. 2 Raman spectrum of HA/MoS_2-Ti6. Source data are provided as a Source Data file.

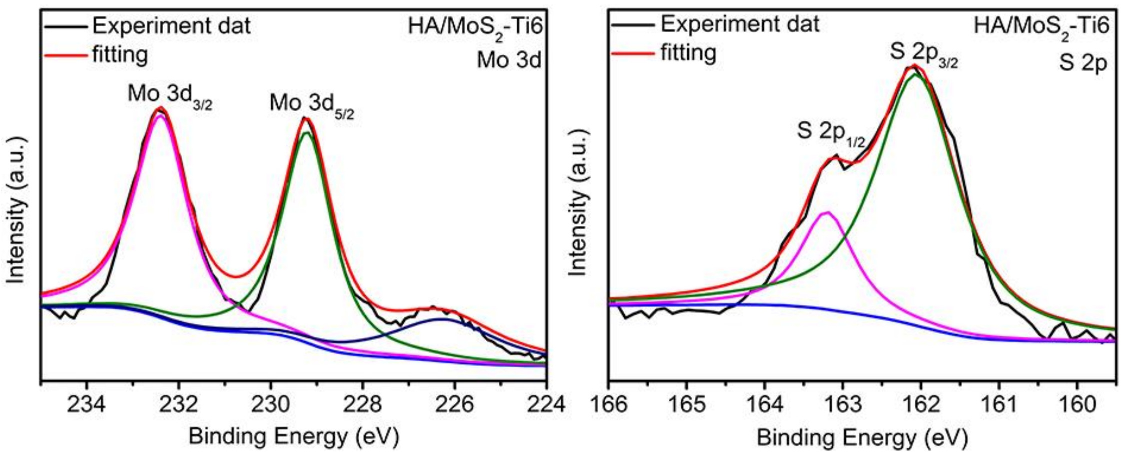

Supplementary Fig. 3 The high-resolution XPS spectra of Mo 3d and S 2p of HA/MoS_2-Ti6. Source data are provided as a Source Data file.

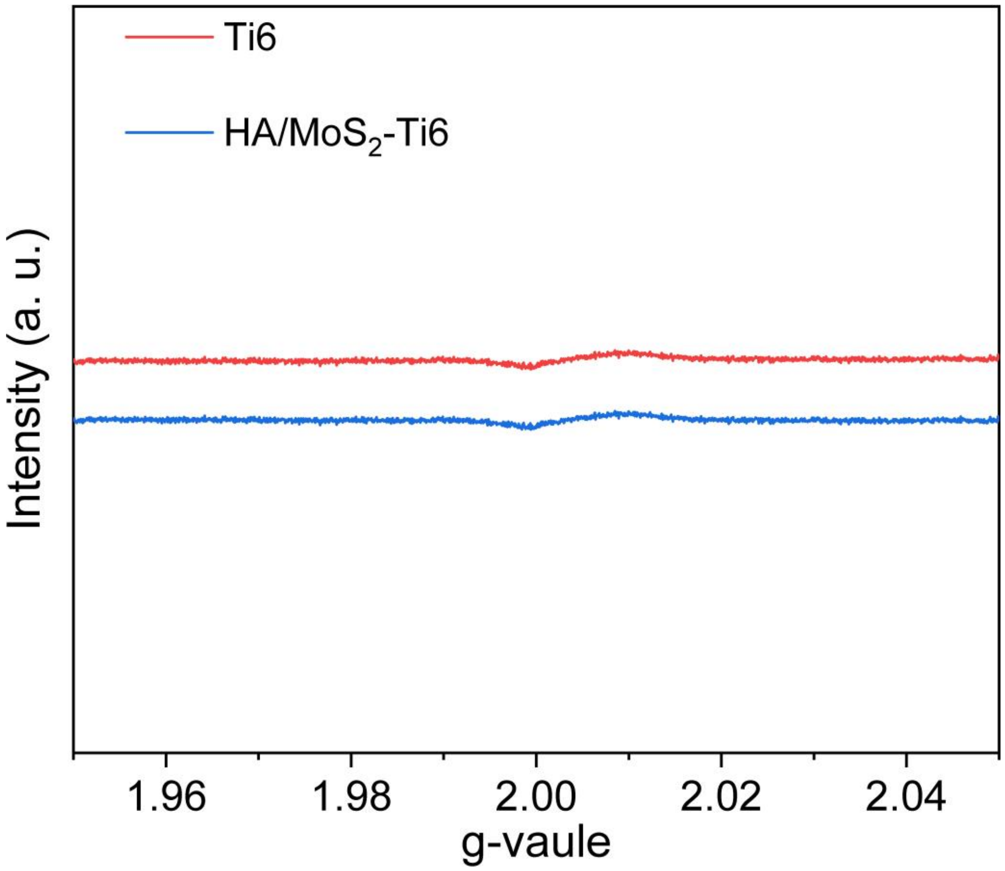

Supplementary Fig. 4 The EPR spectra of Ti6 and HA/MoS_2-Ti6. Source data are provided as a Source Data file.

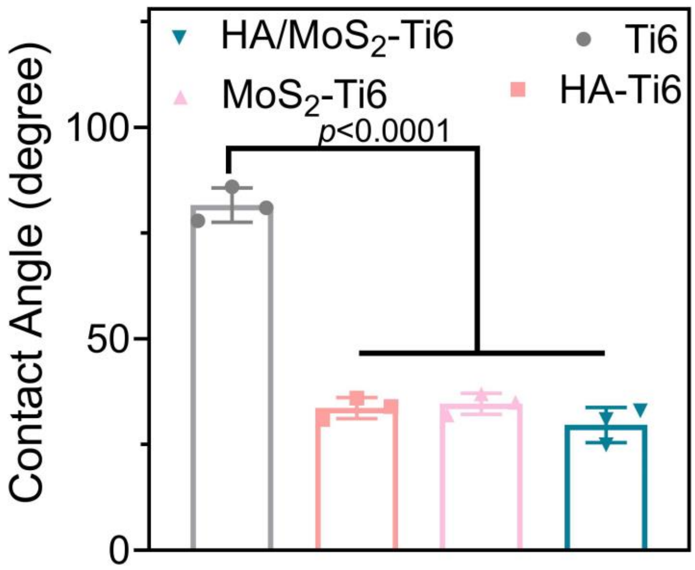

Supplementary Fig. 5 Water contact angle of Ti6, HA-Ti6, MoS_2-Ti6 and HA/MoS_2-Ti6. Data are presented as mean ± standard deviations from a representative experiment. Error bar represents the standard deviation. *p* values were generated by one-way ANOVA with Dunnett's multiple comparison test. (n = 3 independent samples). Source data are provided as a Source Data file.

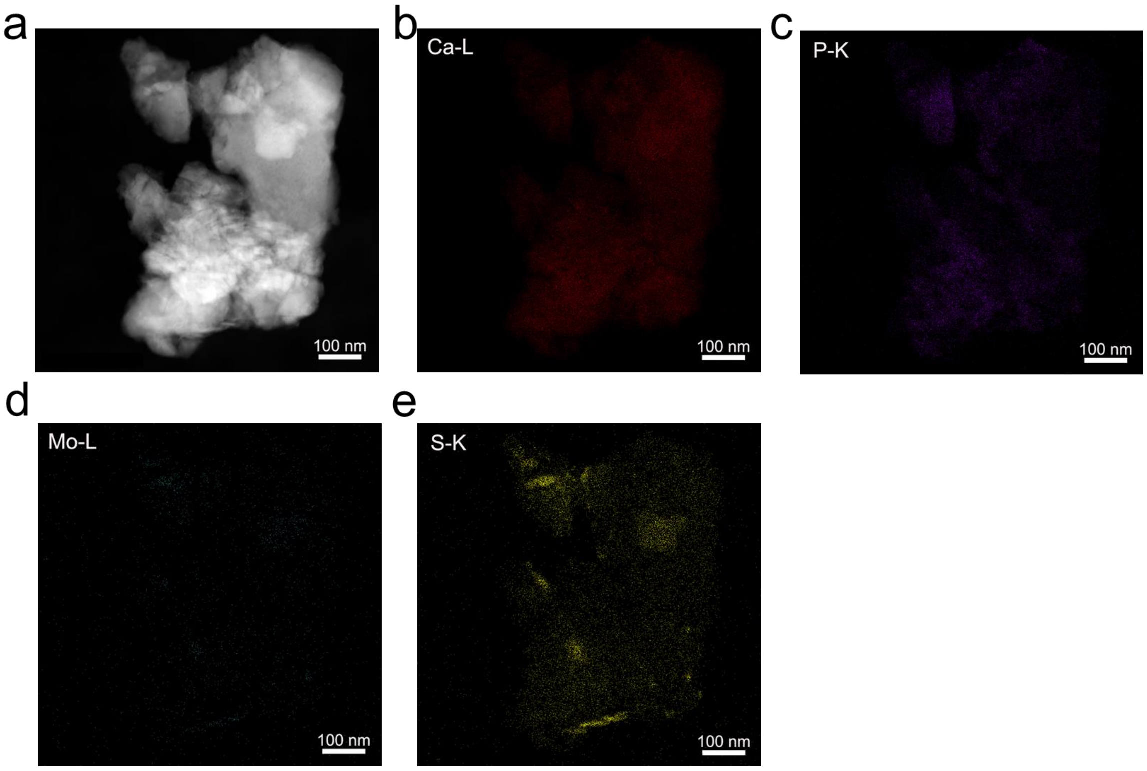

Supplementary Fig. 6 a, The HAADF image of HA/MoS_2. **b-e,** EDS mapping images of HA/MoS_2. (n=3 independent samples).

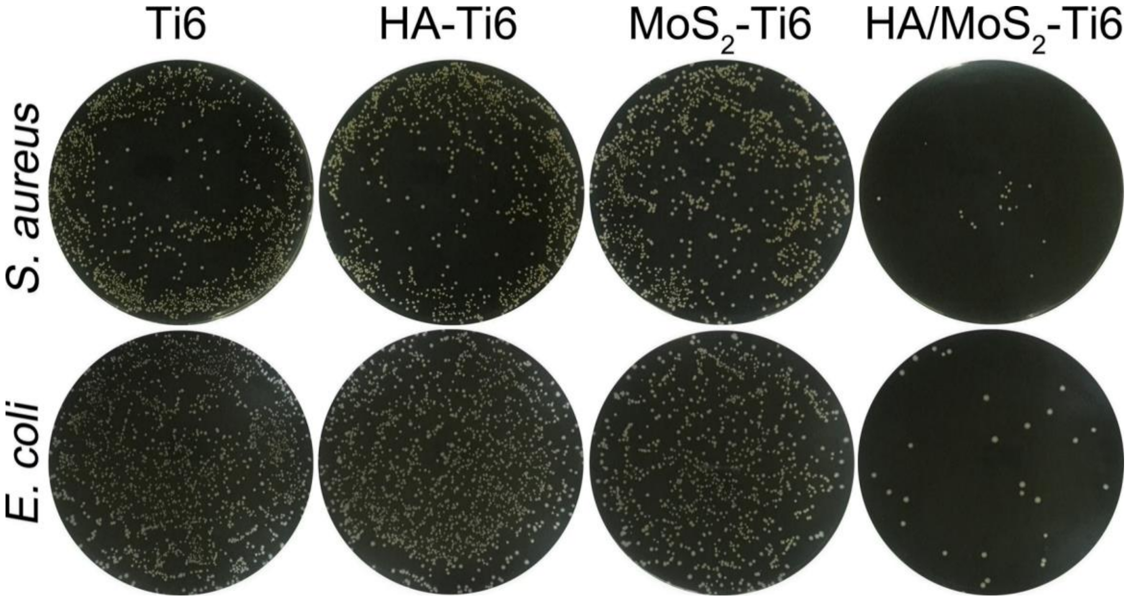

Supplementary Fig. 7 Spread plate results of *S. aureus* and *E. coli* treated with Ti6, HA-Ti6, MoS_2-Ti6, and HA/MoS_2-Ti6.

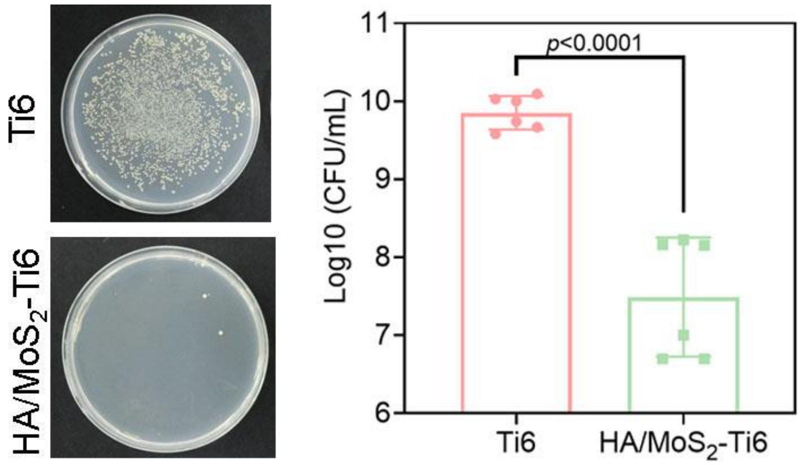

Supplementary Fig. 8 The antibacterial activity of Ti6, and HA/MoS_2-Ti6 against MRSA. Data represent mean ± standard deviations from a representative experiment. Error bar represents the standard deviation. *p* values were generated by t test. (n=6 independent samples). Source data are provided as a Source Data file.

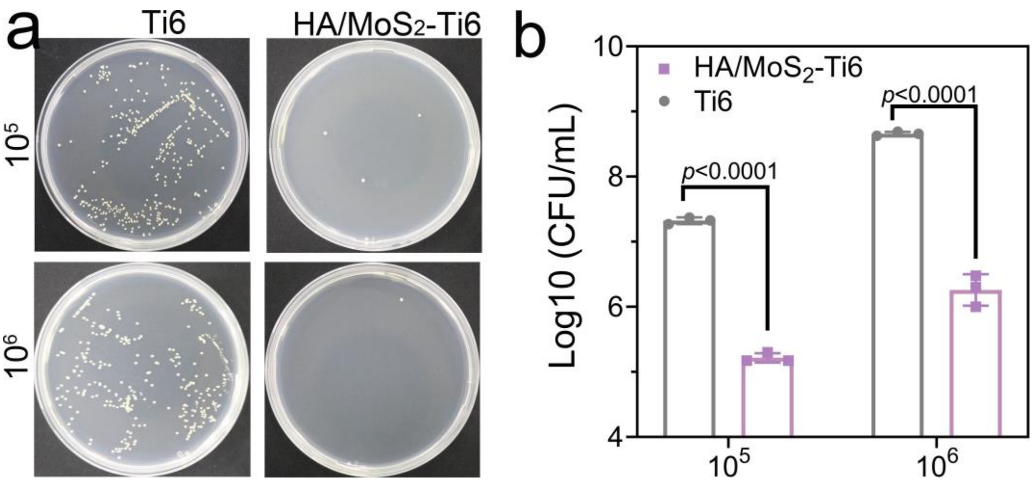

Supplementary Fig. 9 The antibacterial activity of Ti6, and HA/MoS_2-Ti6 against *S. aureus* at different concentration of CFU mL^{-1}. Data represent mean ± standard deviations from a representative experiment. Error bar represents the standard deviation. *p* values were generated by t test. (n=3 independent samples). Source data are provided as a Source Data file.

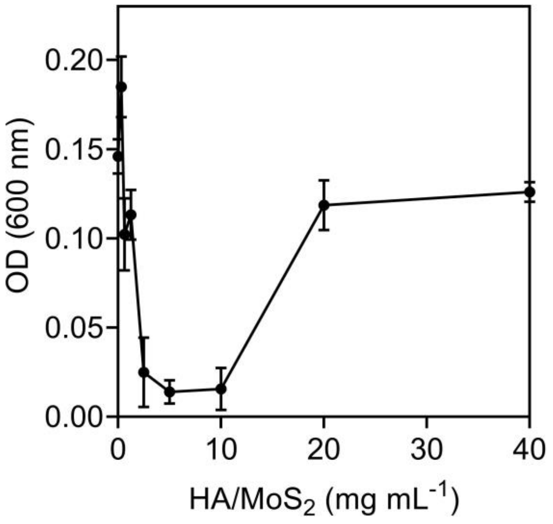

Supplementary Fig. 10 OD_{600} of *S. aureus* co-cultured with HA/MoS_2 under different preparation conditions after culturing for 6 h. Data represent mean ± standard deviations from a representative experiment. Error bar represents the standard deviation. (n=3 independent samples) Source data are provided as a Source Data file.

Method for MIC determination of HA/MoS_2 against *S. aureus*. The OD_{600} value of *S. aureus* co-cultured with HA/MoS_2 under different preparation conditions was not completely linearly related to the concentration of HA/MoS_2, which was related to the formation process of HA/MoS_2-Ti6. The MIC value of HA/MoS_2 was 5 mg mL^{-1}.

On the one hand, the coating formed by high concentration of HA/MoS_2 powers was not ideal due to the limited energy of laser cladding and vulcanization depth. The antibacterial activity of HA/MoS_2 was turned weaker when the concentration of HA/MoS_2 was bigger than 10 mg mL^{-1}. On the other hand, the MIC value of coating was larger than the MIC value of nanoparticles. The contact area between the coating and bacteria was much smaller than the contact area between the nanoparticles and bacteria. And nanoparticles had a larger specific surface area compared with the coating, which could offer more active sites for antibacterial activity.

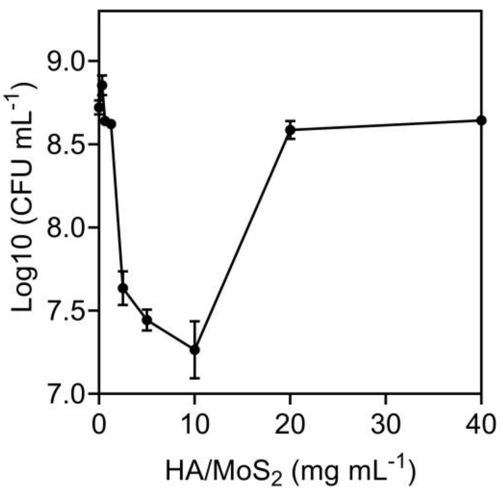

Supplementary Fig. 11 Sample concentration-Log_{10} (CFU mL^{-1}) curves used to determine MBC values. Data represent mean ± standard deviations from a representative experiment. Error bar represents the standard deviation. (n=3 independent samples) Source data are provided as a Source Data file.

Method for MBC determination of *S. aureus*. The Log_{10} (CFU mL^{-1}) value of *S. aureus* co-cultured with HA/MoS_2 under different preparation conditions was not completely linearly related to the concentration of HA/MoS_2, and the MBC value of HA/MoS_2 was 10 mg mL^{-1}. The reason was same as the reason mentioned in MIC part above.

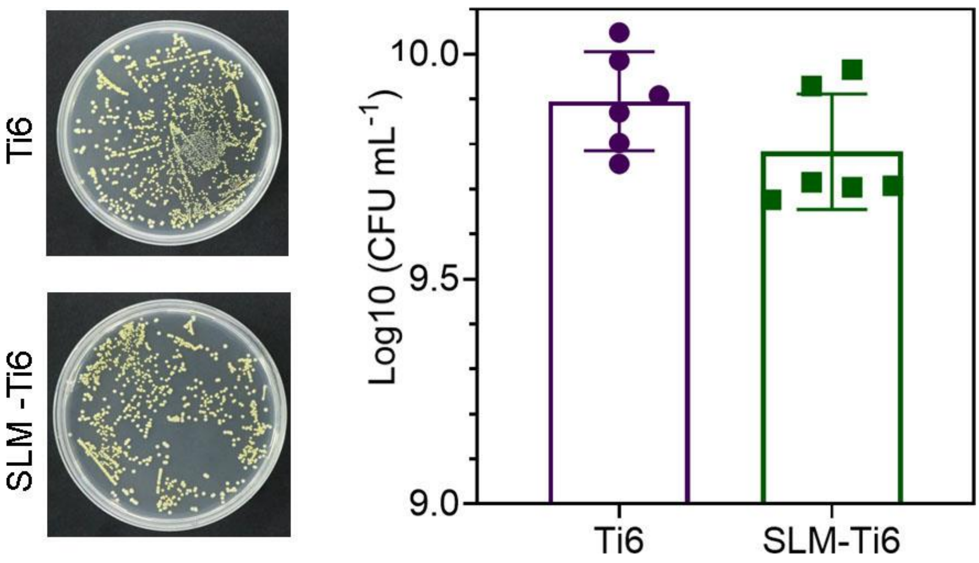

Supplementary Fig. 12 The antibacterial activity of Ti6, and SLM-Ti6 against *S. aureus*. Data represent mean ± standard deviations from a representative experiment. Error bar represents the standard deviation. *p* values were generated by t test. (n=6 independent samples). Source data are provided as a Source Data file.

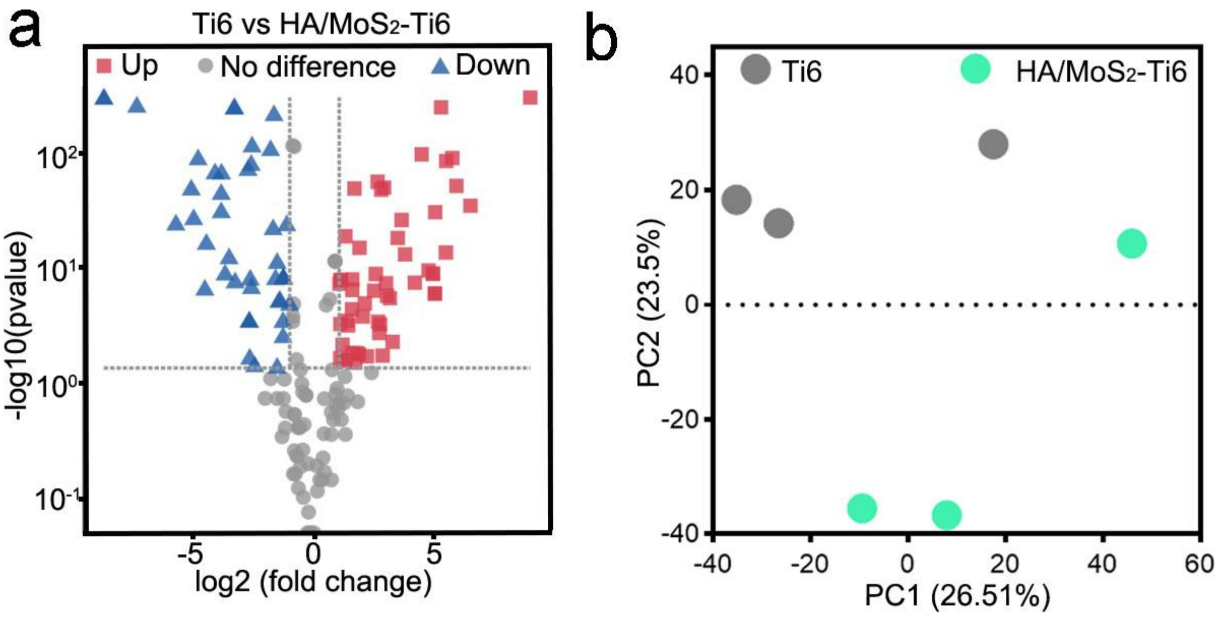

Supplementary Fig. 13 a, Volcano plots of differentially expressed gene for Ti6 vs HA/MoS_2-Ti6. Values are presented as the $\log_2$ of fold change. *p* value was generated by using t test. **b,** PCA plot for Ti6 and HA/MoS_2-Ti6. PCA plot shows PC1 and PC2 for all RNA-seq data of Ti6, and HA/MoS_2-Ti6. The distance between two samples indicates the similarity of different samples. The increasing distance means a growing difference. Source data are provided as a Source Data file.

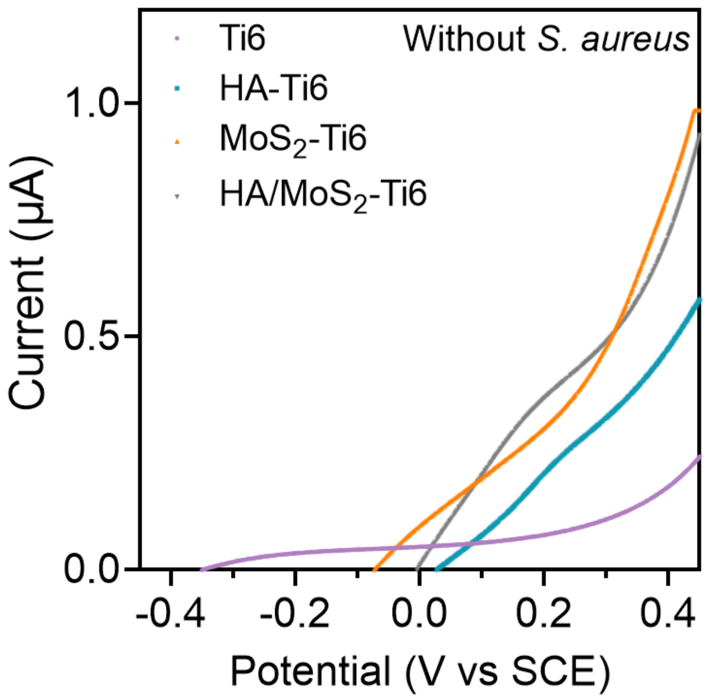

Supplementary Fig. 14 LSV curves of different samples without *S. aureus*. Source data are provided as a Source Data file.

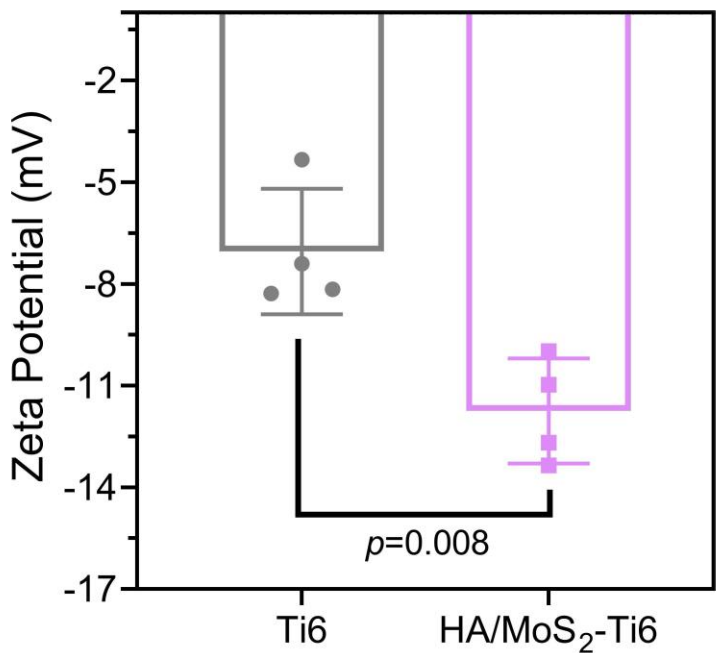

Supplementary Fig. 15 Zeta potential of *S. aureus* after culturing with Ti6 and HA/MoS_2-Ti6. Data represent mean ± standard deviations from a representative experiment. Error bar represents the standard deviation. *p* values were generated by t test. (n=4 independent samples). Source data are provided as a Source Data file.

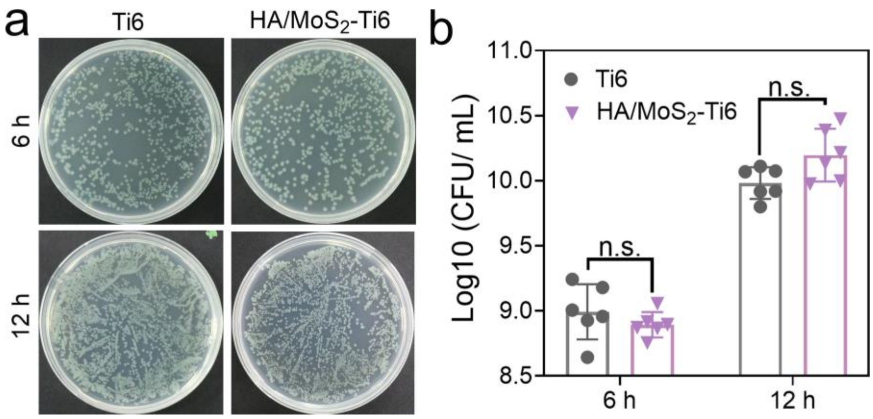

Supplementary Fig. 16 The antibacterial activity of Ti6 and HA/MoS_2-Ti6 against *P. aeruginosa* after culturing for 6 h and 12 h. Data represent mean ± standard deviations from a representative experiment. Error bar represents the standard deviation. *p* values were generated by t test. The n.s. present $p > 0.05$. (n=6 independent samples). Source data are provided as a Source Data file.

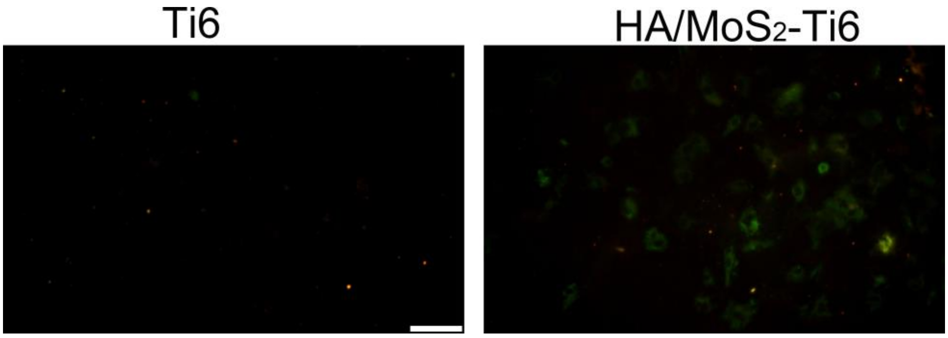

Supplementary Fig. 17 Fluorescence imaging of Ca^{2+} influx in MSCs of Ti6 and HA/MoS_2-Ti6. (n=3 independent samples) Scale bar = 100 μm.

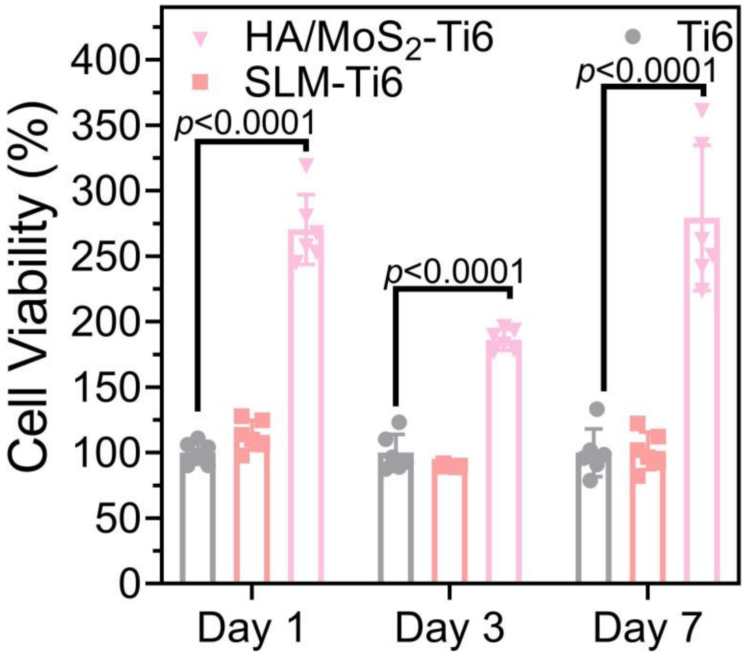

Supplementary Fig. 18 The cell viability of MSCs co-cultured with Ti6, SLM-Ti6 and HA/MoS_2-Ti6 after culturing for 1 day, 3 days, and 7 days. Data represent mean ± standard deviations from a representative experiment. Error bar represents the standard deviation. *p* values were generated by two-way ANOVA with Dunnett's multiple comparison test. (n=6 independent samples). Source data are provided as a Source Data file.

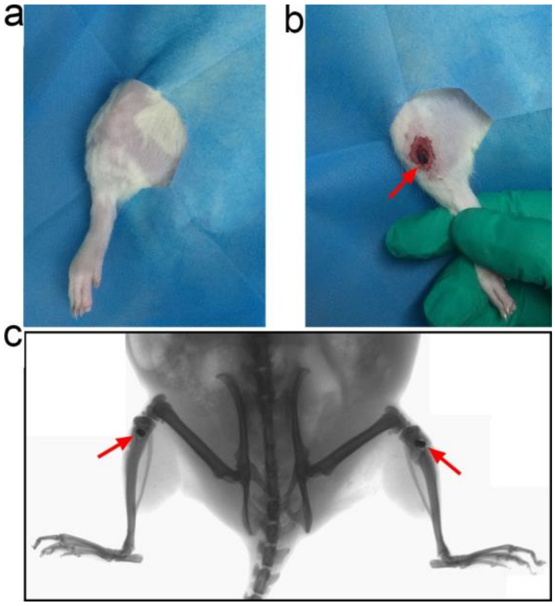

Supplementary Fig. 19 a, The photograph of shaving hair near the tibia. **b,** The photograph of implant location. **c,** X-ray image of titanium rod.

2-SUPPLEMENTARY TABLE

Supplementary Table 1. The primer sequences of the MSCs in the qRT-PCR gene expression analysis.

gene	5’-3’	primers
RUNX2	forward	AATGCCTCCGCTGTTATG
	reverse	CCGGAGTCTATTCACCACCACCTTACT
COL-1	forward	ATGCCGCGACCTCAAGATG
	reverse	TGAGGCACAGACGGCTGAGTA
ALP	forward	AGCGACACGGACAAGAAGC
	reverse	GGCAAAGACCGCCACATC
GAPDH	forward	GCCTCGTCTCATAGACAAGATGGT
	reverse	GAAGGCAGCCCTGGTAACC
