# Supplementary material for: Self-activating anti-infection implant
Source: Nat Commun. 2021 Nov 25;12:6907. doi: 10.1038/s41467-021-27217-4 (PMC8617033; doi:10.1038/s41467-021-27217-4)
Supplement: Supplementary file 3 — Reporting Summary [file 41467_2021_27217_MOESM3_ESM.pdf]

nature research

Corresponding author(s): Shuilin Wu, Chunyong Liang and Xiangmei Liu

Last updated by author(s): Nov 5, 2021

Reporting Summary

Nature Research wishes to improve the reproducibility of the work that we publish. This form provides structure for consistency and transparency in reporting. For further information on Nature Research policies, see Authors & Referees and the Editorial Policy Checklist .

Statistics

For all statistical analyses, confirm that the following items are present in the figure legend, table legend, main text, or Methods section.

n/a	Confirmed	
☐	☒	The exact sample size (*n*) for each experimental group/condition, given as a discrete number and unit of measurement
☐	☒	A statement on whether measurements were taken from distinct samples or whether the same sample was measured repeatedly
☐	☒	The statistical test(s) used AND whether they are one- or two-sided<br>*Only common tests should be described solely by name; describe more complex techniques in the Methods section.*
☒	☐	A description of all covariates tested
☒	☐	A description of any assumptions or corrections, such as tests of normality and adjustment for multiple comparisons
☐	☒	A full description of the statistical parameters including central tendency (e.g. means) or other basic estimates (e.g. regression coefficient) AND variation (e.g. standard deviation) or associated estimates of uncertainty (e.g. confidence intervals)
☐	☒	For null hypothesis testing, the test statistic (e.g. *F*, *t*, *r*) with confidence intervals, effect sizes, degrees of freedom and *P* value noted<br>*Give P values as exact values whenever suitable.*
☒	☐	For Bayesian analysis, information on the choice of priors and Markov chain Monte Carlo settings
☒	☐	For hierarchical and complex designs, identification of the appropriate level for tests and full reporting of outcomes
☒	☐	Estimates of effect sizes (e.g. Cohen's *d*, Pearson's *r*), indicating how they were calculated

Our web collection on statistics for biologists contains articles on many of the points above.

Software and code

Policy information about availability of computer code

Data collection	Commercial software was used to collect data. Microsoft excel 2016
Data analysis	The data were analysed by using Origin 2021, Microsoft Excel 2016, GraphPad Prism 8.0, FastqStat.jar (v0.11.4), RSeQC (v2.6.4) and R package edgeR (v3.24) For image presentation and quantification, Image-pro plus (6.0.0.260) and Adobe Illustrator CS6 was used.

For manuscripts utilizing custom algorithms or software that are central to the research but not yet described in published literature, software must be made available to editors/reviewers. We strongly encourage code deposition in a community repository (e.g. GitHub). See the Nature Research guidelines for submitting code & software for further information.

Data

Policy information about availability of data

All manuscripts must include a data availability statement. This statement should provide the following information, where applicable:

- Accession codes, unique identifiers, or web links for publicly available datasets
- A list of figures that have associated raw data
- A description of any restrictions on data availability

The Source Data underlying Figs. 2d, 2e, 2g-j, 2l, 3b-c, 3e-f, 4a-d, 4f, 5b-c, 5e-f, 5g-h, 5j, 6d, 6f, 6h, 6j, Supplementary Figs. 2, 3, 4, 5, 8b, 9b, 10, 11,12b,14,15,16b,18 can be found in the Source Data File with this paper. The RNA-seq data generated in this study are available on the National Center for Biotechnology Information (NCBI) database under the BioProject PRJNA777398. Other data containing all reported averages in graphs in the figures are provided in the Supplementary Dataset.

Field-specific reporting

Please select the one below that is the best fit for your research. If you are not sure, read the appropriate sections before making your selection.

☒ Life sciences ☐ Behavioural & social sciences ☐ Ecological, evolutionary & environmental sciences

For a reference copy of the document with all sections, see nature.com/documents/nr-reporting-summary-flat.pdf

Life sciences study design

All studies must disclose on these points even when the disclosure is negative.

Sample size	Each finding was confirmed with minimum necessary number such as 1-8 replicates for each experiments. The sample was randomly choose to be determined. These sample sizes are sufficient which have reached statistical significance.
Data exclusions	No data were excluded from the analyses.
Replication	All experiments were performed with independent replicates as described in the figure legends.
Randomization	All samples were randomly allocated into experimental groups.
Blinding	No blinding was applied. The data analyses are based on objectively measurable data, so the investigators were not blinded to allocation during experiments and outcome assessment.

Reporting for specific materials, systems and methods

We require information from authors about some types of materials, experimental systems and methods used in many studies. Here, indicate whether each material, system or method listed is relevant to your study. If you are not sure if a list item applies to your research, read the appropriate section before selecting a response.

Materials & experimental systems

n/a	Involved in the study	
☒	☐	Antibodies
☐	☒	Eukaryotic cell lines
☒	☐	Palaeontology
☐	☒	Animals and other organisms
☒	☐	Human research participants
☒	☐	Clinical data

Methods

n/a	Involved in the study	
☒	☐	ChIP-seq
☒	☐	Flow cytometry
☒	☐	MRI-based neuroimaging

Eukaryotic cell lines

Policy information about cell lines

Cell line source(s)	MSCs cells were obtained from Tongji Hospital (Wuhan, China)
Authentication	No further authentication was done after the cells were obtained from the vendors.
Mycoplasma contamination	All cell lines were tested for mycoplasma contamination. No mycoplasma contamination was found.
Commonly misidentified lines (See ICLAC register)	There were no misidentified cell lines.

Animals and other organisms

Policy information about studies involving animals; ARRIVE guidelines recommended for reporting animal research

Laboratory animals	Male Sprague Dawley rats (each approximately 300-350 g in body weight) were obtained from the Beijing Huafukang Biotechnology Company.
Wild animals	The study did not involve wild animals.
Field-collected samples	The study did not involve samples collected from the field.

Ethics oversight

The study was carried out in accordance with the Guide for the Care and Use of Laboratory Animals of the National Institutes of Health. The ethical aspects of the animal experiment were approved by the Animal Ethical and Welfare Committee (AEWC) of the Institute of Radiation Medicine, Chinese Academy of Medical Sciences (Approval No. YSY-DWLL-2021016).

Note that full information on the approval of the study protocol must also be provided in the manuscript.
